# Inhibiting Glutaminase Reverses Senescence‐Associated Mitochondrial Dysfunction and Skeletal Muscle Decline

**DOI:** 10.1111/acel.70703

**Published:** 2026-10-07

**Authors:** Hamsa Vardini Senthil Kumar, Debanik Choudhury, Pedro Lei, Pihu Mehrotra, Shahryar Shahini, Sydney Swedick, John Toftegaard, Stelios T. Andreadis

**Affiliations:** ^1^ Genetics Genomics and Bioinformatics Program University at Buffalo Buffalo New York USA; ^2^ Department of Chemical and Biological Engineering University at Buffalo Buffalo New York USA; ^3^ Center for Cell, Gene and Tissue Engineering (CGTE) University at Buffalo Buffalo New York USA; ^4^ Department of Biomedical Engineering University at Buffalo Buffalo New York USA; ^5^ Center of Excellence in Bioinformatics and Life Sciences University at Buffalo Buffalo New York USA

## Abstract

Age‐associated loss of skeletal muscle mass and function, also known as sarcopenia, is closely linked to mitochondrial dysfunction. In this study, we report that senescent skeletal muscle myoblasts exhibit elevated activity and expression of the enzyme glutaminase (GLS1), which is mediated by p38 MAPK signaling and leads to intracellular urea accumulation that impairs mitochondrial function. Pharmacological inhibition of GLS1 with CB‐839 reduced urea levels, restored the expression of electron transport chain (ETC) complexes, and improved mitochondrial function. Consistent with our in vitro findings, CB‐839 administration to progeroid mice similarly upregulated ETC complexes and enhanced mitochondrial respiratory capacity in skeletal muscle, leading to improved structural integrity and enhanced muscle strength. Together, these findings suggest that targeting glutamine metabolism via GLS1 inhibition may represent a promising therapeutic strategy to ameliorate age‐related skeletal muscle decline by restoring mitochondrial health.

## Graphical abstract

Pharmacological inhibition of glutaminase with drug CB‐839 restores mitochondrial function in senescent myoblasts and enhances muscle strength and endurance in progeria mice.

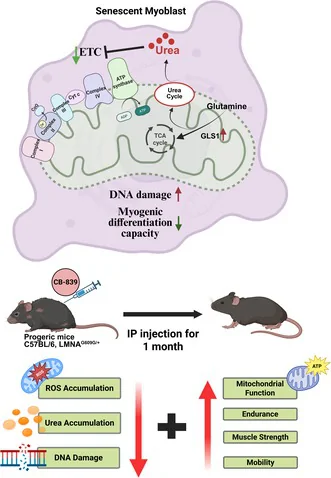

## 1 Introduction

Skeletal muscle, comprising more than 35% of body mass (Xiong et al. 2018), is a vital and highly regenerative organ. Its significance extends beyond facilitating locomotion to actively regulating metabolism, thereby playing a pivotal role in overall health (Stump et al. 2006). Aging is the main cause of sarcopenia, a gradual and persistent decline in skeletal muscle mass and function, leading to diminished mobility and a compromised quality of life (Cruz‐Jentoft and Sayer 2019). The pathogenesis of age‐related muscle wasting involves intricate cellular and molecular mechanisms, characterized by a reduction in fiber cross‐sectional area (Nilwik et al. 2013), alterations in satellite cells (Hikida 2011), and a discernible decline in mitochondrial function (Short et al. 2005). Numerous investigations suggest that modifications in mitochondrial function may indeed constitute a central and crucial factor contributing to muscle quality and function (Figueiredo et al. 2009; Figueiredo et al. 2008; Andreux et al. 2018).

Age‐related mitochondrial dysfunction significantly contributes to skeletal muscle decline, impacting muscle health, aerobic capacity, and overall function between the ages of 30 and 70 (Power et al. 2013). Mitochondria, crucial for cellular energy production and muscle adaptability, undergo age‐related changes, including decreased volume, increased oxidative damage, and reduced respiratory capacity, leading to muscle function impairment, atrophy, and compromised regeneration (Duguez et al. 2002; Chen et al. 2023). Additionally, the decline in the activity of mitochondrial electron transport chain complexes I and IV in aged skeletal muscle is associated with mitochondrial dysfunction and reduced muscle performance (Brierley et al. 1998; Pesce et al. 2001; Nuccio et al. 2024). Preserving mitochondrial health becomes crucial in countering skeletal muscle decline with age, underlining its pivotal role in sustaining overall physical performance.

Hutchinson‐Gilford progeria syndrome, commonly referred to as progeria, is characterized by premature aging resulting from a mutation in the Lamin A (LMNA) gene. A point mutation of cytosine to thymine at position 1824 in exon 11 of the LMNA gene results in the production of a truncated form of lamin A, termed progerin, which accumulates and compromises the integrity of nuclear lamina (Skoczynska et al. 2015). Progerin drives premature aging by inducing DNA damage, histone modifications, and oxidative stress—factors that also contribute to cellular senescence (Wheaton et al. 2017; Cristofalo et al. 2004). LMNA Knock‐In (LAKI) mice exhibit key features of premature aging, including musculoskeletal and cardiovascular abnormalities, and exhibit a markedly shortened lifespan (Ocampo et al. 2016; Paine et al. 2024). Consequently, the progeroid mouse model has gained widespread acceptance as an effective tool for studying aging and assessing potential anti‐aging therapies (Folgueras et al. 2018; Shahini et al. 2021; Rajabian, Ikhapoh, et al. 2023).

Building on our previous findings (Choudhury et al. 2022), this study investigates the interplay between cellular senescence, glutamine metabolism, and mitochondrial function in skeletal muscle myoblasts. Glutamine metabolism plays critical anabolic and bioenergetic roles in skeletal muscle by supporting redox homeostasis and mitochondrial function (Cruzat et al. 2018; Lambertucci et al. 2012). However, emerging evidence suggests that its effects may be multifaceted and context dependent. Senescent cells accumulate in aging tissues, and glutamine metabolism has been shown to support their survival (Johmura et al. 2021). Additionally, we previously demonstrated that increased glutamine catabolism impairs mitochondrial function in senescent mesenchymal stromal cells (MSCs) (Choudhury et al. 2022). However, the effects of glutamine metabolism on senescent skeletal myoblasts and aging skeletal muscle remain poorly understood.

Using in vitro models and a mouse model of accelerated aging, we discovered elevated glutaminase activity and urea accumulation in senescent myoblasts and aged skeletal muscle. Notably, inhibition of this metabolic pathway improved muscle strength and enhanced mitochondrial function both in vitro and in vivo. Collectively, these findings reveal the pro‐aging effects of increased glutamine catabolism and highlight the therapeutic potential of targeting this pathway to mitigate age‐related decline in skeletal muscle and mitochondrial function.

## 2 Materials and Methods

### 2.1 Experimental Animals

Lamin‐A Knocked In (LAKI, C57BL/6, LmnaG609G/G609G) mice were generously provided by Dr. Dudley Lamming at the University of Wisconsin‐Madison School of Medicine, Madison, Wisconsin, USA. For this study, mice of both sex at 10 months of age were employed, chosen at an age when the heterozygous progeria mice (LmnaG609G/+) exhibit evident signs of aging. The mice were accommodated in a temperature‐controlled animal holding room (20.5°–23.9°C) with a 12‐h light/12‐h dark cycle from 6:00 to 18:00 standard time. Food and water were made available ad libitum. Approval of all animal experiments was granted by the Institutional Animal Care and Use Committee (IACUC) at the University at Buffalo.

### 2.2 Treatment of Mice With CB‐839

Heterozygous Progeria mice (LmnaG609G/+) of mixed sex, aged 10 months, received intraperitoneal injections of CB‐839 (10 mg/kg) or vehicle control (5% DMSO in corn oil) three times a week for 1 month, followed by tissue collection.

Skeletal muscle tissues (Tibialis Anterior and Gastrocnemius) were harvested, with a portion of each tissue snap‐frozen in liquid nitrogen for subsequent protein, RNA, or mitochondria isolation. The remaining tissue was immediately immersed in embedding medium (OCT, Sakura Finetek, Torrance, CA) and frozen in 2‐methylbutane (Sigma‐Aldrich) chilled with dry ice. Tissue sections (10 μm thick) were then cut using a freezing microtome (CM1950, Leica), placed on positively charged glass slides (Stellar Scientific, Baltimore, MD), and stored at −80°C.

### 2.3 Cell Culture

Primary human myoblasts from a 68‐year‐old male, 18‐year‐old male, and 17‐year‐old female were purchased from Cook Myosite Inc., Pittsburgh, PA. The cells were expanded in growth medium composed of high‐glucose Dulbecco's modified Eagle's medium (Gibco, Grand Island, NY) supplemented with 20% fetal bovine serum (Atlanta Biologicals, Flowery Branch, GA), bovine serum albumin (0.5 mg/L), epidermal growth factor (10 ng/mL), basic fibroblast growth factor (1 ng/mL), gentamycin (10 μg/mL), and 1% Antibiotic‐Antimycotic. The cells were passed every 4 days before they reached 80% confluency and were cultured on Matrigel (0.1 mg/mL; Corning, NY) coated tissue culture treated flasks. In general, human myoblasts at passage 2–6 were characterized as proliferating young cells (Y). For DNA damage induced senescence model, Y myoblasts were treated with 25 μm etoposide for 24 h, followed by 48 h in fresh culture medium (Eto). Human myoblasts, at passage 11–13, were characterized as replicative induced senescent cells (S), as described previously. For oxidative stress induced senescence model, cells were treated with 200 μM H_2_O_2_ for 48 h, followed by an additional 48 h of 200 μM H_2_O_2_ treatment (H_2_O_2_). To differentiate myoblasts into multinucleated myotubes, myoblasts were seeded on Matrigel (0.1 mg/mL)‐coated dishes and allowed to reach > 90% confluence in GM. Then, the cells were switched to differentiation medium composed of high‐glucose DMEM supplemented with BSA (500 μg/mL) and gentamicin (10 μg/mL) for a period of 5 days.

Mouse myoblasts were isolated from Gastrocnemius muscle of male and female C57BL/6 WT mice or heterozygous progeria mice at the age of 11 months according to our previously established protocol. For differentiation, the cells were allowed to reach 100% confluence (in 2–3 days) and then the medium was switched to differentiation medium that was composed of DMEM with high glucose, 5% horse serum (HS), and 1% AA to promote formation of multinucleated myotubes.

### 2.4 In Vivo Contractile Measurements

Mice were subjected to anesthesia with isoflurane (2% isoflurane, 0.6% oxygen) and subsequently positioned on a heated stage maintained at 37°C using a warm water pump. Eye ointment was applied, and the nose was inserted into the anesthesia device (1.5% isoflurane, 0.6% oxygen) throughout the experiment. To immobilize the knee, a knee clamp affixed to the stage was used, and the leg was secured onto the footplate connected to a servomotor (1300 A: 3‐in‐1 Whole Animal System—Mouse; Aurora Scientific). Two needle electrodes were then inserted into the tibialis anterior (TA) muscle near the knee. The optimal position for muscle contraction was determined by adjusting the distance between the footplate and the knee through single electrical stimulation (25 mA, 0.2 ms pulse width; pre‐optimized conditions). Once the muscle force plateaued, indicating the best contraction position, various forces were measured. Twitch force was assessed with three repetitions of single electrical stimulation (0.2 ms pulse width) at 30 s intervals, taking approximately 2 min. Tetanic force was measured with a 500 ms duration, 0.2 ms pulse width, and a series of frequencies (10, 20, 35, 50, 65, 80, 100, 150, 200 Hz) at 2 min intervals, with a maximum experiment duration of 20 min. After completion, mice were returned to cages with a warming pad and monitored until recovery. Data analysis utilized 611 A Dynamic Muscle Analysis (DMA) software.

### 2.5 Inverted Hang Test

Inverted grip endurance was assessed using an apparatus consisting of a 30 × 30 cm wire grid with 1.4 cm spacing positioned over a 40 cm open‐ended box. Each mouse was placed on the grid, which was then inverted and suspended upside down until it lost grip and fell or reached a maximum test duration of 300 s. Holding impulse of each mouse was calculated by multiplying the hanging time of each mouse by its body weight.

### 2.6 Gait Speed

Gait speed was assessed using a walking‐track apparatus comprising an elevated platform with an approximately 1‐m‐long narrow enclosed channel leading to a darkened enclosure at one end. For each trial, mice were placed at the starting end of the track, and the time required to reach the enclosure was recorded. Three trials were performed, and the two fastest times were averaged to determine the final value.

### 2.7 Western Blot Analysis

For Western Blot analysis, myoblast cells or muscle tissues underwent lysis in RIPA buffer supplemented with 0.1% (w/v) bromophenol blue, 41.67 mM DTT, and a protease inhibitor cocktail. Muscle tissues were homogenized through bead disruption, and protein isolation was conducted at 4°C using the Bullet Blender. The resulting lysates were centrifuged at 18,000*g* for 10 min at 4°C, and the supernatants were collected. The protein content in each sample was determined via a BCA assay. Subsequently, lysates were denatured by incubation at 95°C for 5 min, and 45 μg of proteins were loaded per lane, separated in 4%–20% acrylamide gels (Thermo Fisher Scientific, Waltham, MA) through SDS‐polyacrylamide gel electrophoresis based on molecular weight. Following transfer to nitrocellulose membranes (Bio‐Rad) using the Trans‐Blot Turbo Transfer System (Bio‐Rad, Hercules, California), membranes were blocked for 1 h at room temperature in 2% BSA (for phosphoproteins) or 5% (w/v) non‐fat dry milk (for all other proteins) in blocking buffer. Subsequent overnight incubation at 4°C involved the use of antibodies. Afterward, membranes were exposed for 1 h at room temperature to either Anti‐rabbit IgG HRP linked (Cell Signaling, Cat# 7074; RRID: AB_2099233) or Anti‐mouse IgG HRP linked (Cell Signaling, Cat# 7076; RRID: AB_330924). Finally, protein bands were visualized using horseradish peroxidase‐conjugated secondary antibodies and a chemiluminescence kit (Cell Signaling, Danvers, MA) following the manufacturer's instructions. Chemiluminescent blots were captured using the ChemiDoc Touch Imaging.

### 2.8 Quantitative Real‐Time PCR

Human or mouse myoblast cells were cultured in a 150 mm Petri dish at a density of 3000 cells/cm^2^. Total RNA extraction was performed using the RNeasy Mini Kit (Qiagen, Valencia, CA) following the manufacturer's guidelines. Subsequently, cDNA was synthesized using the Superscript III cDNA Synthesis Kit (Invitrogen) and subjected to real‐time PCR (1 μg per reaction) with the SYBR Green Kit (Bio‐Rad, Hercules, CA). Analysis of qPCR cycles was conducted using Bio‐Rad Software CFX Manager Ver 3.1.

### 2.9 Immunohistochemistry

The tissue sections stored at −80°C underwent three PBS washes to eliminate OCT, followed by fixation and permeabilization with −20°C methanol (Sigma‐Aldrich, St. Louis, MO) for 10 min. Subsequently, the sections were blocked for 1 h with 5% (w/v) goat serum and 2% (w/v) BSA in PBS, followed by Tyramide Blocking Buffer for 1 h and mouse IgG blocking reagent (MOM, Vectors Lab, Burlingame, CA) for 1 h, following the manufacturer's protocol. Overnight incubation at 4°C with primary antibodies diluted in MOM diluent followed. The next day, samples were washed with PBS and stained with Tyramide kit goat anti‐mouse secondary antibody according to the Tyramide kit protocol. Subsequently, the samples were stained with Alexa Fluor 568, 488, or 647 conjugated goat anti‐rabbit or goat anti‐mouse secondary antibodies for 1 h at room temperature.

For immunostaining of cultured cells, fixation occurred for 10 min in 4% paraformaldehyde, followed by permeabilization with 0.1% (v/v) triton X‐100/PBS for 10 min at RT. Blocking was done with 5% (v/v) goat serum in 0.01% (w/v) triton X‐100/PBS at RT for 1 h. Subsequently, samples were immunostained with primary antibodies. The cells were then incubated with Alexa Fluor conjugated goat anti‐mouse or goat anti‐rabbit antibodies (1:200 dilution in blocking buffer, Thermo Fisher Scientific, Waltham, MA) for 1 h at room temperature and counter‐stained with Hoechst 33342 nuclear dye (1:1000 dilution in PBS) for 5 min at RT.

### 2.10 Seahorse Assay

The Seahorse extracellular flux (XFe96) analyzer (Agilent Technologies, Santa Clara, CA) was used to assess oxygen consumption rate (OCR), as a measure of mitochondrial respiration. Proliferating human myoblasts were seeded at 5000 cells/well and their senescent counterparts were seeded at 8000 cells/well, in XFe96 Seahorse culture plates. After 24 h, cells were rinsed and transitioned to Seahorse Base Medium (XF DMEM medium, #103575, Agilent Technologies), supplemented with 10 mM glucose and 1 mM pyruvate. The Mito stress test compounds were prepared in the assay media (180 μL/well) and loaded into the corresponding ports of the hydrated sensor cartridge. Final well concentrations were: 1 μM of Oligomycin (Port A), 1.5 μM FCCP (Port B), 0.5 μM Rotenone & 0.5 μM Antimycin A (Port C). The injection volumes were 20, 22, and 25 μL for Ports A, B, and C respectively. OCR was recorded using the standard seahorse XF measurement protocol, which calls for four measurement cycles one at baseline and after each injection, each cycle consisting of 3 min mixing followed by 3 min measurement. Following completion of the Seahorse run, cells were trypsinized and counted, and the acquired OCR values were normalized to the cell count.

### 2.11 Quantification and Statistical Analysis

Statistical analysis was performed using GraphPad Prism 8–10. Comparisons among more than two samples were performed using one‐way ANOVA followed by Tukey's post hoc test or Dunnett's multiple comparisons test. Unpaired *t*‐tests were used for comparison between two samples. Data are represented as mean ±95% confidence interval (CI) or mean ± SD, as specified in the figure legend. Each experiment was repeated at least three times with triplicate samples per condition to ensure reproducibility.

## 3 Results

### 3.1 Urea Accumulation Impairs Mitochondrial Function of Human Myoblasts

To induce senescence in human myoblasts, we employed three distinct approaches: DNA‐damage induced senescence through etoposide treatment (Moiseeva et al. 2023; Rajabian, Choudhury, et al. 2023) (Figure 1A), oxidative stress (H_2_O_2_) induced senescence (Toussaint et al. 2000; Wang et al. 2013) (Figure 1C), and replicative senescence (Shahini et al. 2021; Rajabian, Ikhapoh, et al. 2023) (Figure 1E). Across all three senescence models, we observed a significant increase in intracellular levels of urea in senescent human myoblasts compared to proliferating non‐senescent myoblasts (Figure 1B,D,F). Given the well‐established association between cellular senescence and mitochondrial dysfunction (Miwa et al. 2022), we hypothesized that urea accumulation may result in impaired mitochondrial function in senescent myoblasts.

**FIGURE 1:**
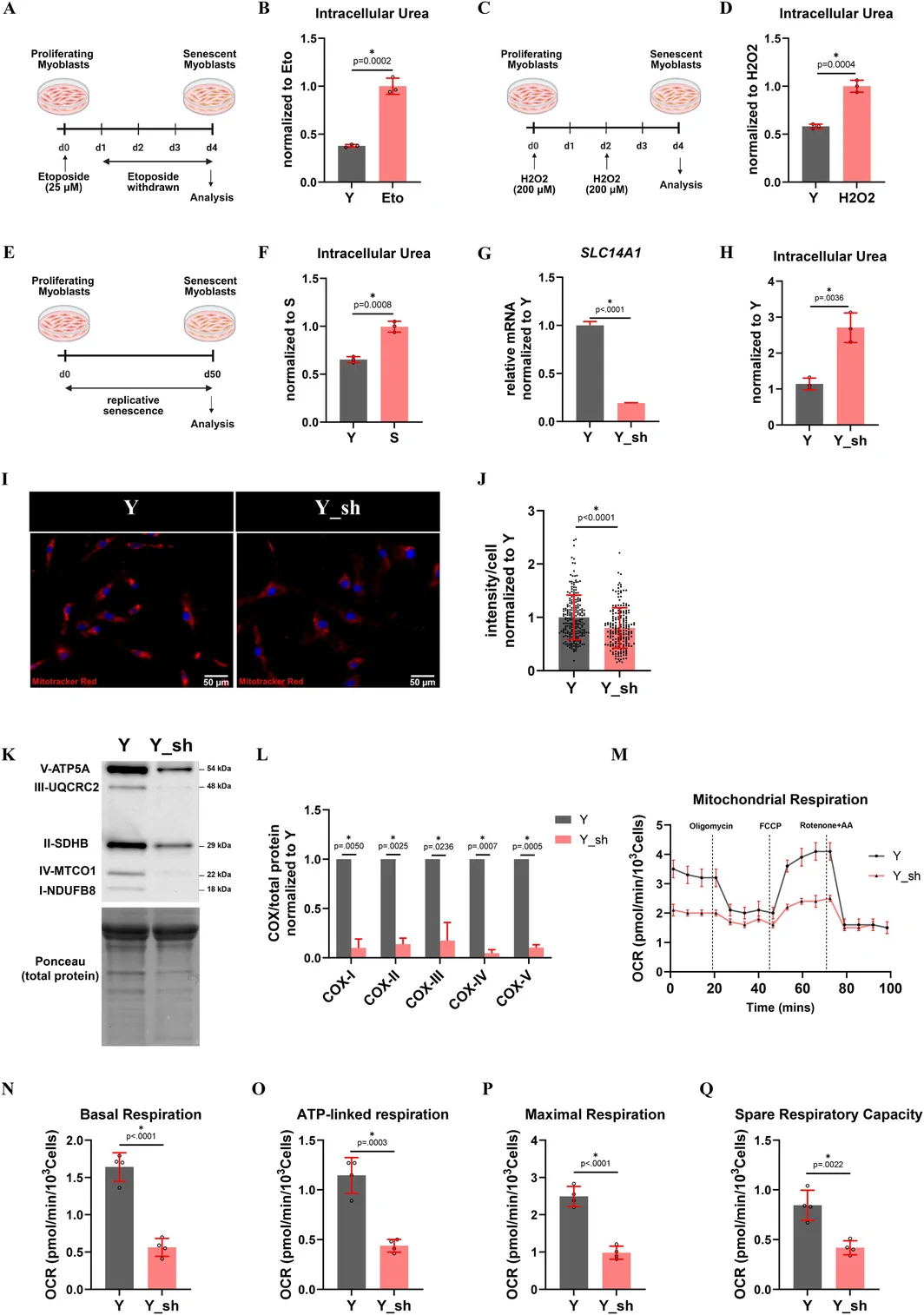
Urea accumulation impairs mitochondrial function and promotes pro‐aging phenotype in senescent human myoblasts. (A) Schematic demonstrating experimental design for inducing senescence in human myoblasts via etoposide treatment. (B) Intracellular urea concentration in young proliferating cells (Y) relative to DNA‐damage induced senescent model (Eto); data shown as fold‐change normalized to Eto and represents mean ± SD, from three independent experiments. (C) Schematic demonstrating experimental design for inducing senescence in human myoblasts via H_2_O_2_ treatment. (D) Intracellular urea concentration in young proliferating cells (Y) relative to oxidative stress induced senescent model (H_2_O_2_); data shown as fold‐change normalized to H_2_O_2_ and represents mean ± SD, from three independent experiments. (E) Schematic demonstrating experimental design for inducing senescence in human myoblasts via replicative senescence (population doubling > 50). (F) Intracellular urea concentration in young proliferating cells (Y) relative to culturally senesced myoblasts (S); data shown as fold‐change normalized to S and represents mean ± SD, from three independent experiments. (G) Quantification of gene expression of SLC14A1 via quantitative RT‐PCR normalized to Y and internally normalized to RPL32; data demonstrating knockdown efficiency and presented as mean ± SD. (H) Intracellular urea level in young proliferating cells (Y) and in Y cells having SLC14A1 knocked down (Y_sh); data shown as fold‐change normalized to Y and represents mean ± SD, from three independent experiments. (I) Representative images of Mitotracker Red live stain; scale bar = 50 μm. (J) Quantification of Mitotracker Red intensity per cell, normalized to Y; data shown as mean ± SD for > 100 cells, from three experiments. (K) Western blot image of mitochondrial complexes probed using an OXPHOS antibody cocktail. Ponceau served as loading control. (L) Quantification of the band intensity of mitochondrial complexes I–V in Y and Y_sh cells. Data shown as mean ± SD. (M) Measurement of oxygen consumption (OCR) using Seahorse extracellular flux analyzer. (N–Q) Calculations of Basal, ATP‐linked, Maximal Respiration, and Spare Respiratory Capacity rates based on OCR data in (M). Data shown as mean ± SD. (A, C, E) Created in BioRender. Andreadis, S. (2026) https://BioRender.com/4x0pnsm. Statistical analysis was performed using unpaired *t*‐test.

To test this hypothesis, we knocked down the urea transporter in proliferating human myoblasts (Figure 1G), forcing young cells (Y) to accumulate urea (Y_sh). Indeed, knockdown of the urea transporter, SLC14A1, resulted in elevated intracellular urea levels (Figure 1H), which was accompanied by reduced mitochondrial membrane potential (Figure 1I,J), and a drastic decrease in the protein levels of mitochondrial electron transport chain complexes (Figure 1K,L). As a result, mitochondrial respiratory activity was markedly impaired in SLC14A1 knock‐down myoblasts, as measured by the oxygen consumption rate (OCR) using a Seahorse analyzer (Figure 1M), revealing reduced basal and ATP‐linked respiration (Figure 1N,O), maximal respiration (Figure 1P), and spare respiratory capacity (Figure 1Q). Concomitantly, we observed increased DNA damage, as indicated by an increase in γ‐H2AX foci (Figure S1A,B), and increased SA‐β‐Gal activity (Figure S1C,D), two well‐established hallmarks of cellular senescence. Collectively, these findings suggest that intracellular urea accumulation leads to mitochondrial dysfunction and myoblast senescence.

### 3.2 Glutaminolysis Inhibition Enhances Mitochondrial Function and Decreases DNA Damage in Senescent Human Myoblasts

Our previous work showed that senescent human mesenchymal stromal cells (MSCs) and myofibroblasts from Hutchinson‐Gilford progeria syndrome exhibited increased reliance on amino acids, particularly glutamine (Choudhury et al. 2022), prompting us to investigate the potential link between increased urea levels observed in senescent human myoblasts and glutamine metabolism. Indeed, we observed elevated expression of GLS1, the enzyme catalyzing glutamine deamination, at both the mRNA and protein levels across all three models of myoblast senescence: etoposide‐induced (Figure S2A–C), H_2_O_2_‐induced (Figure S2D–F), and replication‐induced senescence (Figure S2G–I). To investigate the relationship between glutamine metabolism and urea accumulation in senescent myoblasts, we examined whether inhibiting glutaminase activity could mitigate intracellular urea accumulation following the experimental protocol as depicted in Figure 2A. Treatment with CB‐839, a selective GLS1 inhibitor, for 5 days resulted in a dose‐dependent reduction in GLS activity (Figure 2B) and urea concentration (Figure 2C), with a significant reduction in intracellular urea observed even at the lowest dose tested (0.5 μM). No further reduction in urea levels was observed at higher CB‐839 doses (1 and 2 μM), but cell viability was adversely affected (Figure 2D). Therefore, subsequent experiments evaluating the effects of CB‐839 treatment on senescent myoblasts were conducted at the effective dose of 0.5 μM.

**FIGURE 2:**
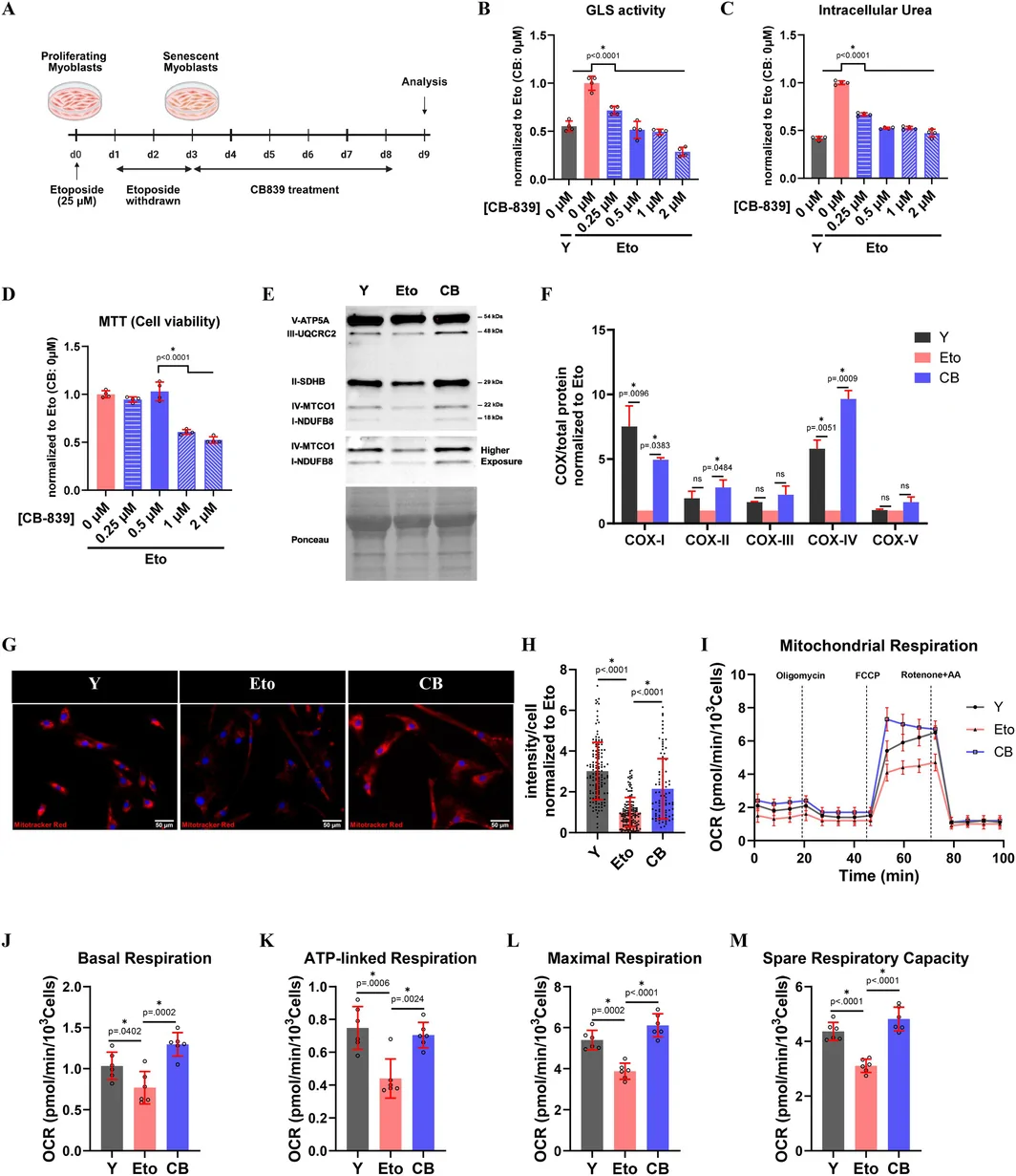
GLS1 inhibition improves mitochondrial function in senescent human myoblasts. (A) Schematic demonstrating the experimental design of CB‐839 treatment in etoposide induced senescent myoblasts. Created in BioRender. Andreadis, S. (2026) https://BioRender.com/hwduzyn (B) Measurement of GLS activity in senescent myoblasts after treatment with different dosages of CB‐839; data shown as fold change normalized to Eto (CB: 0 μM) and represents mean ± SD. (C) Intracellular urea level after treatment of senescent myoblasts (Eto; orange bar) with different dosages of CB‐839; data shown as fold change normalized to Eto (CB: 0 μM) and represents mean ± SD. (D) Effect of different dosages of CB‐839 treatment on cell viability was measured by MTT assay; data shown as fold change normalized to Eto (CB: 0 μM) and represents mean ± SD, from four independent experiments. (E) Western blot image of mitochondrial complexes probed using an OXPHOS antibody cocktail in proliferating cells (Y), senescent myoblasts (Eto), and CB‐839 treated senescent myoblasts (CB). Ponceau served as loading control. (F) Quantification of the band intensity of mitochondrial complexes I–V. Data shown as mean ± SD and normalized to Eto for each complex. (G) Representative images of Mitotracker Red live stain; scale bar = 50 μm. (H) Quantification of Mitotracker Red intensity per cell, normalized to Eto; data shown as mean ± SD for > 100 cells. (I) Measurement of oxygen consumption (OCR) using Seahorse extracellular flux analyzer. (J–M) Calculations of Basal Respiration, ATP‐linked Respiration, Maximal Respiration, and Spare Respiratory Capacity rates based on OCR data in (I) and presented as mean ± SD. Cell Model: 68‐year‐old male subject. Statistical analysis was performed using one‐way ANOVA followed by Tukey's multiple comparisons.

GLS1 inhibition restored the levels of the enzymes in the electron transport chain (ETC), specifically COX‐I, COX‐II, and COX‐IV (Figure 2E,F) and significantly increased mitochondrial membrane potential (Figure 2G,H). In addition to the etoposide‐induced senescence model, restoration of mitochondrial membrane potential following GLS1 inhibition was also confirmed in replicatively senescent myoblasts (Shahini et al. 2021; Rajabian, Choudhury, et al. 2023) (Figure S2J–M). Consistent with these findings, GLS1 inhibition enhanced mitochondrial respiration of senescent cells, as indicated by increased basal, ATP‐linked, maximal, and spare respiratory capacity (Figure 2I–M). CB‐839 treatment also led to decreased γH2AX foci in senescent cells, indicating reduced DNA damage (Figure S2N–O). Importantly, the increase in mitochondrial function and reduction in DNA damage were consistently observed in senescent cells from multiple donors (Figure S3A–R). In contrast, GLS1 inhibition did not alter the mitochondrial membrane potential in young proliferating cells (Figure S3S–T). Notably, GLS1 inhibition did not affect SA‐β‐Gal activity in either etoposide‐induced (Figure S2P–Q) or H_2_O_2_‐induced senescent myoblasts (Figure S2R,S). Together, these findings demonstrate the potential of GLS1 inhibition to reduce urea accumulation, improve mitochondrial function, and alleviate senescence‐associated DNA damage.

### 3.3 GLS1 Expression in Senescent Myoblasts Is Upregulated by p38 Signaling

To better understand the signaling pathways affecting GLS1 expression, we investigated the relationship between glutaminase inhibition and the MAPK pathways, specifically focusing on ERK, JNK, and p38 (Figure 3A). These pathways have been previously linked to age‐associated mitochondrial dysfunction (Javadov et al. 2014; Anerillas et al. 2020). Additionally, our prior work identified JNK as a regulator of GLS1 in senescent stromal cells (Choudhury et al. 2022), prompting us to investigate whether MAPK signaling similarly regulates GLS1 in senescent myoblasts. Among the MAPK pathways examined, pharmacological inhibition of p38 using SB203580 led to the most pronounced decrease in GLS1 mRNA (Figure 3B) and protein levels (Figure 3E,F). Inhibiting p38 also reduced the mRNA levels of both the GLS1 isoforms, KGA and GAC (Figure 3C,D). Two additional p38 inhibitors, SB202190 and Ralimetinib dimesylate, showed a similar reduction in GLS1 mRNA and protein levels (Figure S4A–E). Additionally, p38 inhibition led to significant upregulation of all the mitochondrial electron transport chain proteins. For some of them, namely, COX‐I, COX‐IV, and COX‐V, p38 inhibition was more effective than inhibition of the other two MAPK pathways (Figure 3G–L). Furthermore, inhibiting p38 resulted in enhanced mitochondrial respiration (Figure 3M); at the highest dose (10 μM), the p38 inhibitor restored both basal (Figure 3N) and ATP‐linked respiration (Figure 3O), while also significantly increasing maximal respiration (Figure 3P) and spare respiratory capacity (Figure 3Q). Collectively, these results suggest that, in senescent myoblasts, p38 signaling promotes GLS1 expression, leading to impaired mitochondrial function.

**FIGURE 3:**
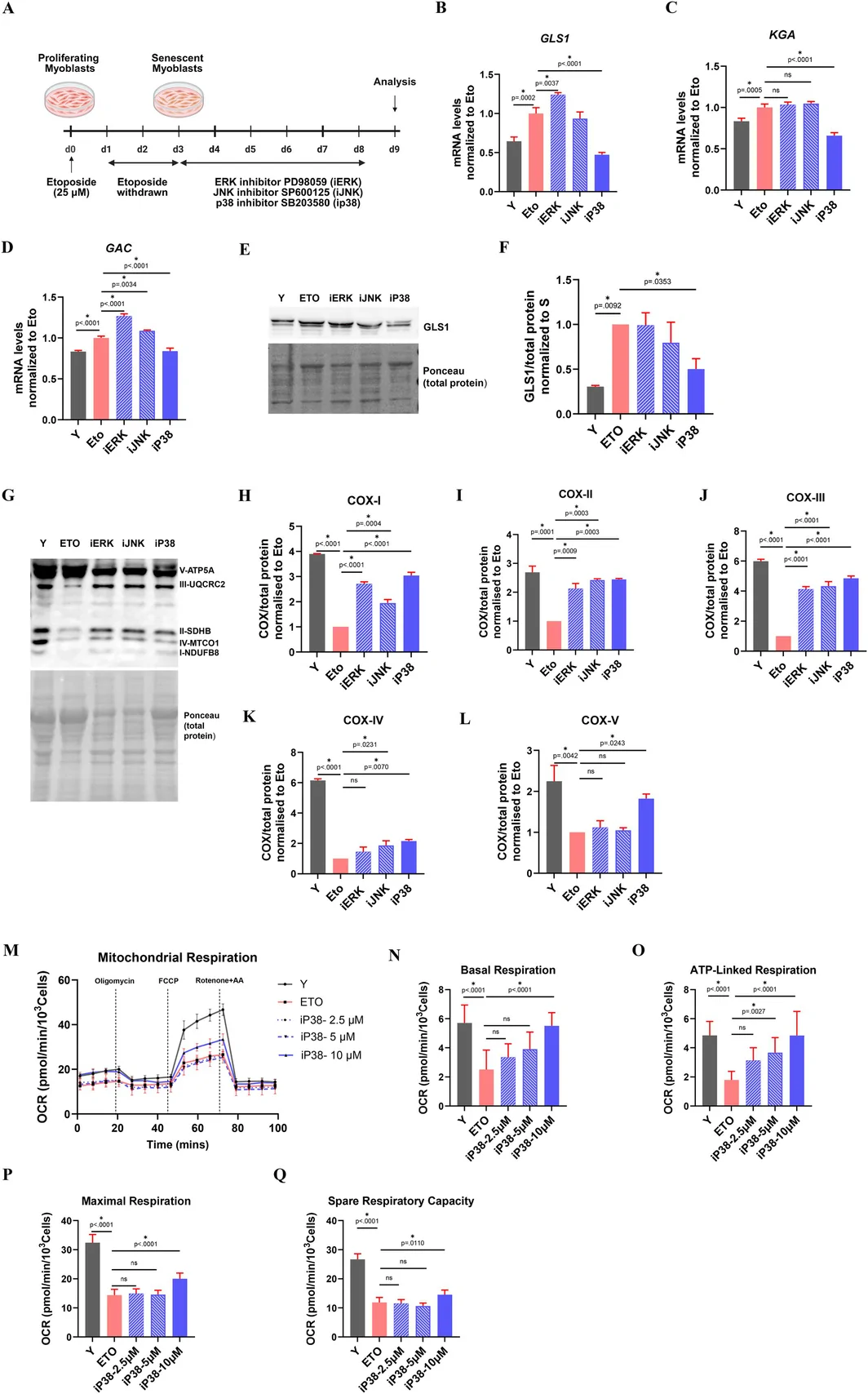
Inhibition of P38 activity decreases levels of GLS1 and enhances mitochondrial health in senescent cells. (A) Schematic demonstrating experimental design of MAPK inhibition in etoposide treated senescent cells; ERK inhibitor: PD98059, JNK inhibitor: SP600125, p38 inhibitor: SB203580 were used at a dose of 10 μM for a period of 5 days. Created in BioRender. Andreadis, S. (2026) https://BioRender.com/jl1a9ds. (B–D) Measurement of mRNA levels of GLS1, KGA, GAC, respectively; normalized to Eto; data shown as mean ± SD. (E) Western blot image of GLS1; ponceau served as loading control. (F) Quantification of the band intensity of GLS1. Data shown as mean ± SD and normalized to Eto. (G) Western blot image of mitochondrial complexes probed using OXPHOS antibody cocktail; ponceau served as loading control. (H–L) Quantification of the band intensity of mitochondrial complexes I–V. Data shown as mean ± SD and normalized to Eto for each complex. (M) Measurement of oxygen consumption (OCR) in senescent myoblasts treated with different dosages of p38 inhibitor for a period of 5 days, using Seahorse extracellular flux analyzer. (N–Q) Calculations of Basal Respiration, ATP‐linked Respiration, Maximal Respiration, and Spare Respiratory Capacity rates based on OCR data in (M) and presented as mean ± SD. All statistical tests were assessed by one‐way ANOVA followed by Tukey's test.

### 3.4 GLS1 Inhibition Improves the Skeletal Muscle Function in Progeria Mice

Next, we evaluated the impact of glutaminase (GLS1) inhibition in a murine model of accelerated aging, specifically the heterozygous progeria mice (LmnaG609G/+ or LAKI). These mice exhibit aging phenotypes emerging progressively from 6 to 10 months of age (Shahini et al. 2021; Zaghini et al. 2020). Intraperitoneal injections of CB‐839 were administered three times a week over a period of 1 month, following our previous protocol (Choudhury et al. 2022) (Figure 4A). Compared to wild‐type (WT) controls, the gastrocnemius (GA) muscle of LAKI mice exhibited elevated glutaminase activity and urea concentration, both of which were significantly reduced following 1 month of CB‐839 treatment (LAKI‐CB) (Figure 4B,C). Furthermore, the GA muscle from CB‐839 treated progeria mice showed decreased reactive oxygen species (ROS) levels (Figure 4D) with a concomitant increase in mitochondrial electron transport chain complexes COX‐I, COX‐II, and COX‐IV (Figure 4E,F). Because mitochondrial dysfunction is a hallmark of skeletal muscle atrophy (Chen et al. 2023) and GLS1 inhibition increased mitochondrial complex abundance in progeroid skeletal muscle, we next assessed whether GLS1 inhibition mitigated age‐associated defects in muscle structure and function (Larsson et al. 2019). Treatment of LAKI mice with CB‐839 resulted in a significant increase in the cross‐sectional area of myofibers as compared to untreated control LAKI mice (Figure 4G,H). The effect on myofiber size prompted us to examine whether GLS1 inhibition affected the expression of Pax7, a marker of satellite cells. Remarkably, we observed a significant increase in the number of Pax7^+^ cells in the LAKI‐CB as compared to the untreated control LAKI mice (Figure 4I,J).

**FIGURE 4:**
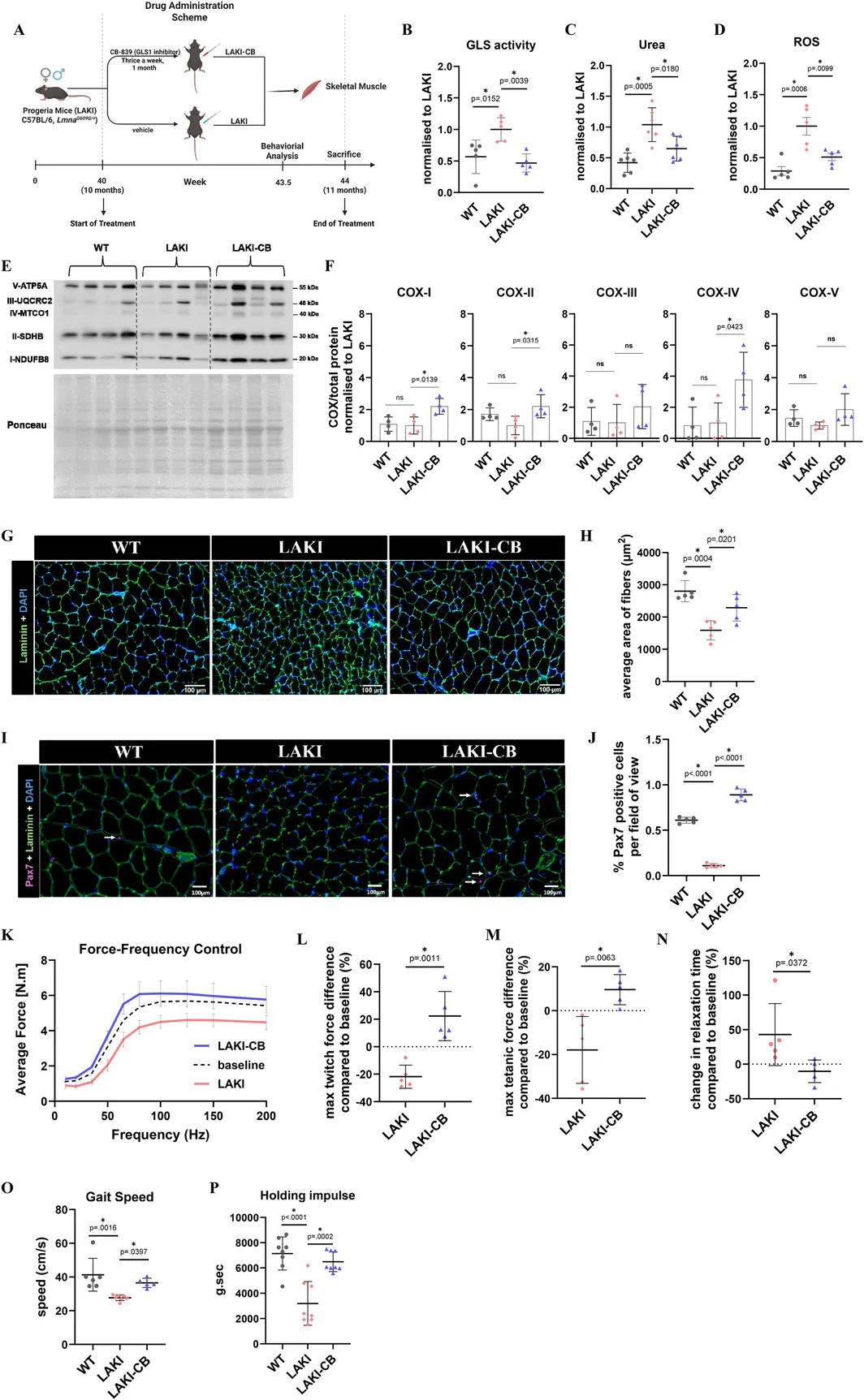
CB‐839 treatment in progeria mice decreases GLS1 activity and intra‐tissue urea concentration and increases mitochondrial complex protein expression. (A) Schematic showing drug administration scheme. Created in BioRender. Andreadis, S. (2026) https://BioRender.com/t2cmy49. (B) Measurement of GLS activity of gastrocnemius (GA) muscle of mice (Wild type: WT; Progeria control: LAKI, Progeria mice treated with CB‐839: LAKI‐CB); data shown as fold change normalized to LAKI and represents mean ± SD (*n* = 3 male and 2 female). (C) Measurement of urea levels of GA muscle; data shown as fold change normalized to LAKI and represents mean ± SD (*n* = 3 male and 3 female). (D) Measurement of ROS level in GA muscle tissue; data shown as fold change normalized to LAKI and represents mean ± SD (*n* = 3 male and 2 female). (E) Western blot image of mitochondrial complexes probed using a rodent OXPHOS antibody cocktail; ponceau served as loading control. (F) Quantification of the band intensity of mitochondrial complexes I–V. Data shown as mean ± SD and normalized to LAKI for each complex (*n* = 2 male and 2 female). (G) Laminin immunostaining (green) of Tibialis Anterior (TA) muscle; scale bar = 100 μM. (H) Quantification of fiber size; data represents mean ± SD from *n* = 3 male and 2 female animals per cohort. (I) Pax7 immunostaining (red) of TA muscle co‐stained with Laminin (green); scale bar = 100 μM. (J) Quantification of percentage of Pax7 positive cells per field of view; data represents mean ± SD from *n* = 3 male and 2 female animals per cohort. (K–N) Effect of CB‐839 treatment on twitch force (L), tetanic force (M) and relaxation time (N) as a function of stimulation frequency in dorsiflexor muscle in situ; data represents mean ± SD (*n* = 3 male and 2 female). (O) Gait speed analysis (average of top two of three times to move 1 m) (WT: *n* = 6, LAKI: *n* = 7, LAKI‐CB: *n* = 5; *n* = 2 female per group) and (P) holding impulse (300 s set as cut‐off) (*n* = 8; *n* = 3 female per group) in WT, LAKI and LAKI‐CB mice; data represents mean ± SD. Each bubble in the dot plot represents one mouse. Statistical analysis was performed using one‐way ANOVA followed by Tukey's test (B–D, H, J, P), Dunnett's test (O, F), and unpaired *t*‐test (L–N).

Additionally, we investigated whether the augmented fiber size was accompanied by enhanced muscle strength (Nilwik et al. 2013). To this end, we utilized a force transducer to measure twitch force, maximum isometric tetanic force, and the force‐frequency relationship generated by the dorsiflexor muscles in live mice. While control LAKI mice exhibited a progressive decline in muscle strength, no such decline was observed in LAKI‐CB mice following a month of treatment (Figure 4K). In comparison to the control group, a marked increase above the baseline was evident in both the twitch force (Figure 4L) and tetanic force (Figure 4M) in the LAKI‐CB group, indicating improved muscle strength. Finally, the relaxation time of the LAKI‐CB dorsiflexor muscles remained close to the baseline and significantly lower than LAKI controls (Figure 4N), indicating faster relaxation. To determine whether these improvements in muscle strength translated into whole‐animal mobility, we employed two additional physical assessment tests, gait speed and holding impulse. Remarkably, LAKI‐CB mice showed marked improvements in both gait speed (Figure 4O) and inverted hang time (Figure 4P). These results suggest that GLS1 inhibition using CB‐839 preserves muscle structure and strength, thereby improving physical performance under conditions of accelerated aging.

### 3.5 Myoblasts From CB‐839 Treated Mice Demonstrate Improved Mitochondrial Function and Myogenic Differentiation Capacity

Following treatment with CB‐839, we isolated myoblasts from the GA muscle of untreated and CB‐839 treated progeria mice (*n* = 5) using our published protocol (Shahini et al. 2018) (Figure 5A). Immunostaining for desmin demonstrated nearly 100% positivity, confirming a pure myoblast population (Figure S5A,B). Interestingly, myoblasts isolated from the skeletal muscle of LAKI‐CB mice retained reduced GLS1 activity (Figure 5B) and decreased intracellular urea content (Figure 5C). Diminished urea accumulation was associated with elevated mitochondrial membrane potential (Figure 5D,E) and improved mitochondrial respiratory chain activity (Figure 5F–J), with no significant change in mitochondrial content (Figure S5C). Consistent with our in vitro findings with human myoblasts, myoblasts isolated from CB‐839 treated animals showed a decreased fraction of γH2AX positive cells, indicating reduced DNA damage (Figure S5D,E). Furthermore, myoblasts isolated from CB‐839 treated LAKI mice demonstrated reduced mRNA levels of inflammatory cytokines IL1α, IL1β, and CCL8 (Figure S5F–I).

**FIGURE 5:**
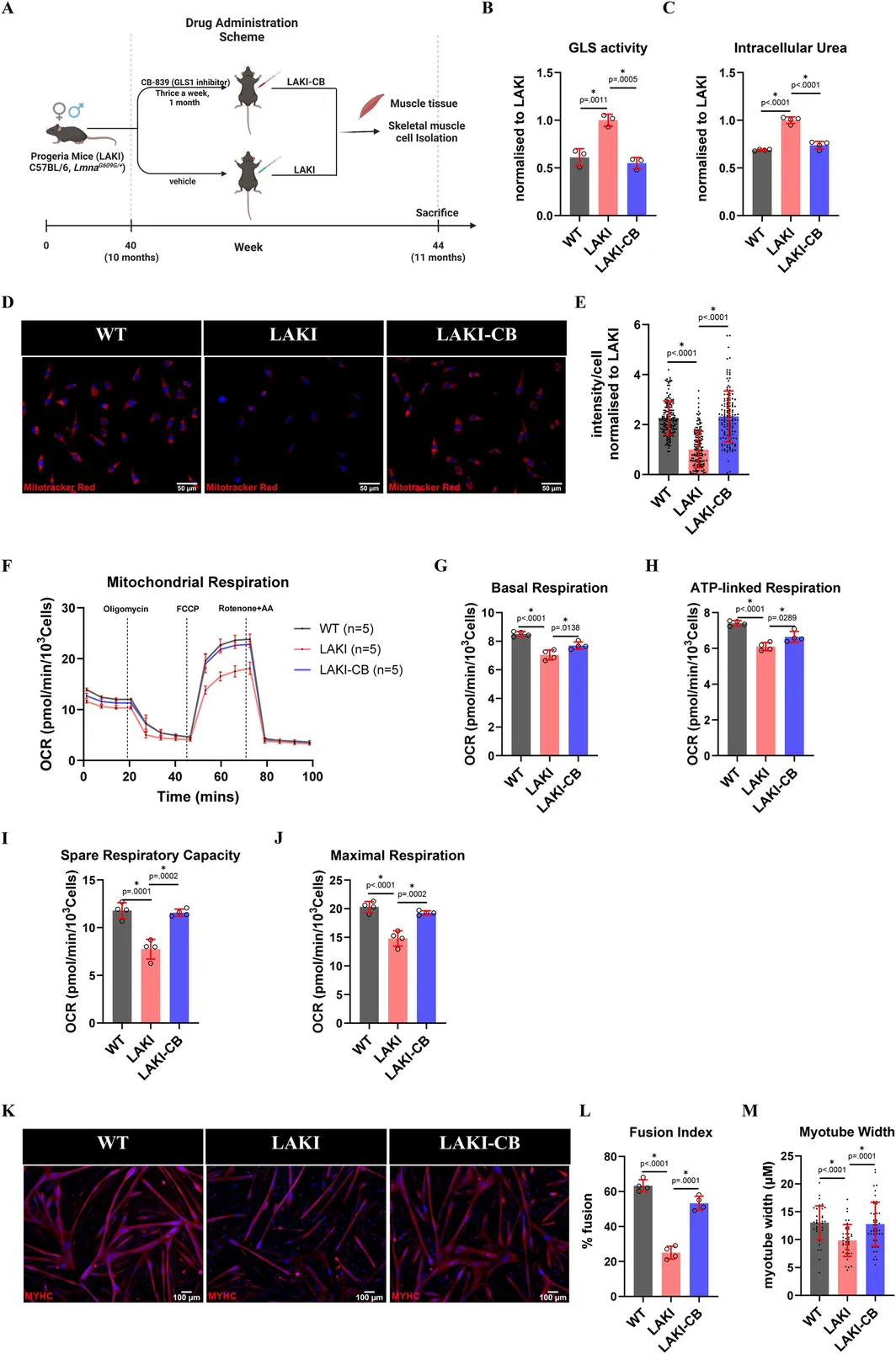
Isolated mouse myoblasts from CB‐839 treated LAKI mice demonstrate improved mitochondrial function and differentiation capacity. Myoblasts were isolated from LAKI, CB‐treated LAKI (LAKI‐CB), and wild‐type mice of same age (WT). From each group of mice, five (*n* = 3 male and 2 female) gastrocnemius muscle (GA) tissues were pooled together and used for myoblast isolation. (A) Schematic showing drug administration scheme. Created in BioRender. Andreadis, S. (2026) https://BioRender.com/t2cmy49. (B) Measurement of GLS activity; normalized to LAKI and data shown as mean ± SD. (C) Intracellular urea concentration, normalized to LAKI; data shown as mean ± SD. (D) Representative images of Mitotracker Red live stain; scale bar = 50 μm. (E) Quantification of Mitotracker Red intensity per cell, normalized to LAKI; data shown as mean ± SD for > 100 cells. (F) Measurement of oxygen consumption (OCR) using Seahorse extracellular flux analyzer. (G–J) Calculations of Basal Respiration, ATP‐linked Respiration, Maximal Respiration, and Spare Respiratory Capacity rates based on OCR data. (K) Immunostaining for MHC (red) of myotubes formed by WT, LAKI, and LAKI‐CB myoblasts. Nuclei were stained with DAPI (blue). (L) Quantification of fusion index (number of myonuclei/total number of nuclei X 100%) and (M) average width of myotubes formed by the three groups. Data are presented as mean ± SD. Statistical analysis was performed using one‐way ANOVA followed by Tukey's multiple comparisons test.

Given that CB‐839 treatment restored mitochondrial function and reversed DNA damage, we further explored its potential to restore the myogenic differentiation potential of LAKI myoblasts. In line with previous studies, LAKI myoblasts demonstrated impaired ability to form myotubes (Figure 5K) (Rajabian, Ikhapoh, et al. 2023). The fusion index (percentage of nuclei in myotubes) and myotube width were significantly decreased in LAKI myotubes compared to wild‐type (WT) myotubes (Figure 5K–M). Intriguingly, myoblasts isolated from CB‐839‐treated LAKI mice exhibited improved myogenic differentiation capacity, as evidenced by increased fusion index (Figure 5L) and myotube width (Figure 5M). These results demonstrate that targeting GLS1 with CB‐839 can effectively mitigate key aging‐related defects in murine progeric muscle.

## 4 Discussion

In this study, we demonstrate that skeletal muscle cell senescence is associated with elevated intracellular urea accumulation, which arises, at least in part, from increased glutamine catabolism driven by upregulated glutaminase 1 (GLS1) expression and activity. Urea accumulation impairs mitochondrial function, an effect that can be mitigated by pharmacological inhibition of GLS1 using the small‐molecule inhibitor CB‐839. GLS1 inhibition reduces intracellular urea levels, restores mitochondrial function, and reverses several hallmarks of skeletal muscle aging both in vitro and in vivo.

Previous work demonstrated that glutaminase inhibition can induce senolysis through intracellular acidification, selectively eliminating senescent cells (Johmura et al. 2021). Although higher doses of CB‐839 (> 2 μM) induced significant cell death in senescent myoblasts, the lower dose used in this study (0.5 μM) significantly reduced GLS1 activity without inducing senescent cell death. Under these conditions, CB‐839 markedly decreased intracellular urea levels, accompanied by reduced DNA damage and improved mitochondrial function. Importantly, CB‐839 had no significant effect on mitochondrial membrane potential in non‐senescent proliferating myoblasts, supporting that its beneficial effects are specific to the senescent state. These findings suggest that GLS1 inhibition acts through a senescence‐associated mechanism rather than a donor age‐specific effect. Interestingly, SA‐β‐gal activity was not reduced by CB‐839 treatment, suggesting that low‐dose CB‐839 restores mitochondrial function in vitro and muscle function in vivo through a mechanism independent of senolytic activity. These findings warrant further investigation into the therapeutic potential and underlying mechanisms of GLS1 inhibition.

To this end, we identified p38 MAPK to be an upstream regulator of GLS1 in senescent myoblasts. The p38 MAPK family, comprising the α, β, γ, and δ isoforms, is well recognized for its roles in muscle physiology and pathology, including muscle atrophy, insulin resistance, and regulation of atrophy‐associated genes such as MURF1 and Atrogin‐1 (Bengal et al. 2020; Yuasa et al. 2018). Pharmacological inhibition of p38 using SB203580 or Ralimetinib dimesylate effectively decreased GLS1 expression and enhanced mitochondrial bioenergetics in senescent myoblasts, suggesting that p38 may be a critical modulator of glutamine metabolism and mitochondrial function in aging muscle cells. Interestingly, our previous work in senescent mesenchymal stem cells (MSCs) revealed that JNK signaling, rather than p38, played a dominant role in regulating GLS1 expression and activity, leading to enhanced glutaminolysis, increased glutamate and TCA cycle intermediates, and the accumulation of urea as a metabolic byproduct (Choudhury et al. 2022). Together, these findings highlight a conserved link between MAPK signaling and GLS1‐mediated intracellular urea accumulation and mitochondrial dysfunction across cell types, while also revealing the cell‐type‐specific regulation of GLS1 by distinct MAPK pathways, with p38 emerging as a prominent regulator in senescent myoblasts and JNK in senescent MSCs.

Similar to the in vitro findings with senescent human myoblasts, elevated GLS1 expression and urea levels were observed in myoblasts isolated from progeric mice. These progeric myoblasts exhibited compromised mitochondrial bioenergetics, a well‐known factor contributing to reduced myogenic fusion capacity (Santanasto et al. 2016; Woo et al. 2018). Interestingly, myoblasts isolated from CB‐839‐treated progeria mice exhibited reduced GLS1 activity and lower urea accumulation, suggesting that in vivo GLS1 inhibition affected myogenic cells within muscle tissue, consistent with the effects observed in myoblasts treated with CB‐839 in vitro. The persistence of these effects following cell isolation and culture suggests a durable, cell‐intrinsic response to in vivo GLS1 inhibition, at least in part by preventing urea accumulation and intracellular inflammation. These cells exhibited improved mitochondrial bioenergetics and enhanced myogenic differentiation capacity, as evidenced by an increased ability to form myotubes in culture. Of note, no change in mitochondrial DNA content (mtDNA/nDNA ratio) was observed in CB‐839‐treated progeroid mice, suggesting that the overall mitochondrial mass remained unchanged. Thus, the improvement in mitochondrial bioenergetics likely reflects enhanced functional capacity of the existing mitochondrial pool rather than increased mitochondrial abundance. The mechanisms through which GLS1 inhibition improves mitochondrial quality and enhances electron transport chain (ETC) function warrant further investigation.

Loss of muscle mass with aging, also referred to as sarcopenia, is a complex process characterized by the convergence of multiple factors including dysregulated signaling pathways (Pascual‐Fernandez et al. 2020), chronic inflammation (Dalle et al. 2017), muscle denervation (Chai et al. 2011), compromised mitochondria (Calvani et al. 2013), and disrupted proteostasis (Paez et al. 2023). As central regulators of skeletal muscle metabolism, mitochondria play a critical role in maintaining muscle integrity. Dysfunctions in mitochondria have been strongly linked to muscle fiber atrophy (Hyatt et al. 2019) and is a well‐established contributor to the decline in muscle strength and motor performance observed in aging individuals (Tian et al. 2022; Tyrrell et al. 2015). For instance, maximal mitochondrial ATP production is inversely correlated with fatigue during walking tests (Santanasto et al. 2016), while elevated mitochondrial respiration rates in permeabilized muscle fibers have been associated with higher walking speed in older adults (Coen et al. 2013). Consistent with these observations, our study found that CB‐839 treatment in progeric mice increased mitochondrial ETC complex expressions and increased muscle fiber size and increased muscle strength. These findings suggest that improved mitochondrial bioenergetics, driven by GLS1 inhibition, contributes to muscle structural integrity and strength. Muscle mitochondrial dysfunction is also associated with poor motor function in aging. In summary, our study uncovers a critical link between glutamine metabolism, urea accumulation, and mitochondrial dysfunction in skeletal muscle aging. We propose that enhanced GLS1 activity, driven in part by p38 MAPK signaling, contributes to bioenergetic deficits in senescent and aging muscle cells. Pharmacological inhibition of GLS1 not only mitigates these defects but also improves muscle structure and function in a progeric mouse model.

These findings support the broader concept that amino acid metabolism plays a critical role in muscle aging. We have previously shown that ectopic expression of *Nanog* restores muscle regenerative capacity by inhibiting methionine metabolism (Rajabian, Ikhapoh, et al. 2023), an amino acid pathway linked to aging. Additional studies have demonstrated that restricting certain amino acids, such as branched‐chain amino acids (BCAAs), can extend lifespan in mice and improve mitochondrial respiratory capacity and glucose sensitivity in individuals with Type 2 diabetes (Trautman et al. 2022; Karusheva et al. 2019). In contrast, certain amino acids like proline (Choudhury et al. 2024) and taurine (Singh et al. 2023) have been reported to enhance mitochondrial function and alleviate age‐related hallmarks. These observations suggest that excessive amino acid metabolism may contribute to aging or age‐related disorders, while individual amino acids exert distinct effects: some accelerating aging, while others providing protective, anti‐aging benefits.

Although our study included both male and female LAKI mice, we did not assess whether GLS1 inhibition produces sex‐dependent effects on mitochondrial function and muscle strength. Given the well‐established sex‐specific differences (Nuccio et al. 2024) in muscle aging and mitochondrial bioenergetics, this remains an important area for future investigation. Future studies investigating sex‐specific responses and the long‐term effects of GLS1 inhibition on lifespan and healthspan will be important for defining the broader therapeutic potential of this approach. Notably, an important aspect of our study is the use of CB‐839 (Telaglenastat), a glutaminase inhibitor that has been clinically investigated as a cancer therapeutic. CB‐839 targets glutamine metabolism in tumors, where glutaminolysis is upregulated to sustain rapid cell proliferation (Yang et al. 2017; Wicker et al. 2021). Our findings raise the possibility that repurposing CB‐839 or related glutaminase inhibitors currently under clinical development for oncology could offer a promising strategy to counteract sarcopenia and preserve skeletal muscle healthspan, warranting further investigation.

## Author Contributions

Experiments were planned and designed by H.V.S.K., D.C., and S.T.A. Experimental data were generated and collected by H.V.S.K., D.C, P.L, P.M., S. Shahini, S. Swedick and J.T. Data analysis and interpretation involved D.C., H.V.S.K. and S.T.A. Writing and critical editing of the manuscript was done by H.V.S.K, D.C. and S.T.A.

## Funding

This work was supported by National Institute on Aging, R01 AG068250.

## Conflicts of Interest

The authors declare no conflicts of interest.

## Supporting information


**Figure S1:** Urea accumulation leads to upregulation of senescence hallmarks in human myoblasts. (A) Immunostaining for phosphorylated form of histone H2AX (γ‐H2AX), a measure of DNA damage, scale bar = 50 μm. Y_sh denotes human myoblasts in which urea transporter, SLC14A1 was knocked down. (B) Quantification of γ‐H2AX intensity per cell, normalized to Y; data shown as mean ±95% CI for > 100 cells, from three experiments. (C) Representative images of SA‐β‐gal staining in Y and Y_sh myoblasts. (D) Percentage of senescent cells shown by SA‐β‐gal staining and quantification; data presented as mean ± SD, from four experiments. Statistical analysis was performed using unpaired *t*‐test.
**Figure S2:** Senescent myoblasts demonstrate increased GLS1 expression. DNA damage‐induced senescence (etoposide): (A) Quantification of *GLS1* gene expression via qRT‐PCR normalized to Eto; data shown as mean ± SD. (B, C) Immunostaining for GLS1, scale bar = 100 μm and quantification of GLS1 intensity per cell normalized to Eto; data shown as mean ± SD for > 100cells. Oxidative stress‐induced senescence (H_2_O_2_): (D) Quantification of *GLS1* gene expression via quantitative RT‐PCR normalized to H_2_O_2_; data shown as mean ± SD. (E, F) Immunostaining for GLS1, scale bar = 100 μm and quantification of GLS1 intensity per cell normalized to H_2_O_2_; data shown as mean ± SD for > 100cells. Replicative senescence model: (G) Quantification of *GLS1* gene expression via quantitative RT‐PCR normalized to S; data shown as mean ± SD. (H, I) Immunostaining for GLS1, scale bar = 100 μm and quantification of GLS1 intensity per cell normalized to S; data shown as mean ± SD for > 100cells. (A–I) Human myoblasts from 68‐years‐old male subject was used. (J, K) Representative images of Mitotracker Red live stain; scale bar = 100 μm and quantification of Mitotracker Red intensity per cell, normalized to S (replicative senescence model in 18‐years‐old male subject); data shown as mean ± SD for > 100 cells. (L, M) Representative images of Mitotracker Red live stain; scale bar = 100 μm and quantification of Mitotracker Red intensity per cell, normalized to S (replicative senescence model; myoblasts from 17‐years‐old female donor); data shown as mean ± SD for > 100 cells. (N, O) Immunostaining for γ‐H2AX, scale bar = 50 μm in Y, Eto and CB‐839 treated (CB) human myoblasts and quantification of γ‐H2AX intensity per cell, normalized to Eto; data shown as mean ± SD for > 100 cells. (P, Q) Representative images of SA‐β‐gal staining and quantification of percentage of senescent cells in etoposide‐induced senescent model; data presented as mean ± SD. (R, S) Representative images of SA‐β‐gal staining and quantification of percentage of senescent cells in H_2_O_2_‐induced senescent model; data presented as mean ± SD. Statistical analysis was performed by using unpaired *t*‐test (A, C, D, F, G, I) and one‐way ANOVA followed by Tukey's test (K, M, O, Q, S).
**Figure S3:** GLS1 inhibition improves mitochondrial function and reduces DNA damage in senescent human myoblasts. (A–I) Human myoblasts from 18 years old male subject was induced to senescence via etoposide. (A) Representative images of Mitotracker Red live stain; scale bar = 100 μm. (B) Quantification of Mitotracker Red intensity per cell, normalized to Eto; data shown as mean ± SD for > 100 cells. (C) Measurement of oxygen consumption (OCR) using Seahorse extracellular flux analyzer. (D–G) Calculations of Basal Respiration, ATP‐linked Respiration, Maximal Respiration, and Spare Respiratory Capacity rates based on OCR data in (C) and presented as mean ± SD. (H, I) Immunostaining for γ‐H2AX (scale bar = 100 μm) and quantification of γ‐H2AX intensity per cell, normalized to Eto; data shown as mean ±95% CI for > 100 cells. (J–R) Human myoblasts from 17 years old female donor were induced to senescence via etoposide. (J) Representative images of Mitotracker Red live stain; scale bar = 100 μm. (K) Quantification of Mitotracker Red intensity per cell, normalized to Eto; data shown as mean ± SD for > 100 cells. (L) Measurement of oxygen consumption (OCR) using Seahorse extracellular flux analyzer. (M–P) Calculations of Basal Respiration, ATP‐linked Respiration, Maximal Respiration, and Spare Respiratory Capacity rates based on OCR data in (L) and presented as mean ± SD. (Q, R) Immunostaining for γ‐H2AX (scale bar = 100 μm) and quantification of γ‐H2AX intensity per cell, normalized to Eto; data shown as mean ±95% CI for > 100 cells. (S, T) Proliferating myoblasts (Y) were treated with CB‐839 for 4 days (Y_CB). (S) Representative images of Mitotracker Red live stain; scale bar = 20 μm. (T) Quantification of Mitotracker Red intensity per cell, normalized to Y; data shown as mean ± SD for > 100 cells. Statistical analysis was performed by using one‐way ANOVA followed by Tukey's test (B, D–G, I, K, M–P, R) and unpaired *t*‐test (T).
**Figure S4:** Inhibition of p38 activity with additional inhibitors decrease mRNA and protein levels of GLS1. (A–C) Measurement of mRNA levels of KGA, GAC and GLS1 respectively; normalized to Eto; data shown as mean ± SD. p38: p38 inhibitor SB203580, p38_1: p38 inhibitor LY2228820 (Ralimetinib dimesylate), p38_2: p38 inhibitor SB202190; Eto cells were treated with the p38 inhibitors at a dose 10 μM for a period of 5 days. (D) Western blot of GLS1; ponceau served as loading control. (E) Quantification of the total band intensity of the two isoforms KGA and GAC. Data shown as mean ± SD and normalized to Eto. Statistical tests were assessed by one‐way ANOVA followed by Dunnett's test (A–C, E).
**Figure S5:** Mouse myoblasts isolated from CB‐839 treated mice show decreased DNA damage. (A, B) Immunostaining and quantification of desmin in isolated mouse myoblasts from WT, LAKI and LAKI‐CB mice, scale bar = 50 μm; data shown as mean ± SD for > 100 cells (C) Quantitative real‐time PCR quantification of mtDNA relative to nDNA (mtDNA/nDNA) as measured by primers specific to mitochondrial gene MT‐TL1 and nuclear gene β2M; data presented as mean ± SD. (D, E) Immunostaining for γ‐H2AX, scale bar = 100 μm and quantification of γ‐H2AX intensity per cell, normalized to LAKI; data shown as mean ± SD for > 100 cells. (F–I) Quantification of gene expression of inflammatory cytokines via quantitative RT‐PCR normalized to LAKI. Data represents mean ± SD. All statistical tests were performed by one‐way ANOVA followed by Tukey's test.

## Data Availability

All data reported in this paper will be shared by the lead contact upon request. This paper does not report original code. Any additional information to reanalyze the data reported in this paper may be directed to and will be fulfilled by lead contact Stelios T. Andreadis (sandread@buffalo.edu).
